# Assessment of the announced North Korean nuclear test using long-range atmospheric transport and dispersion modelling

**DOI:** 10.1038/s41598-017-07113-y

**Published:** 2017-08-18

**Authors:** Pieter De Meutter, Johan Camps, Andy Delcloo, Piet Termonia

**Affiliations:** 1 Belgian Nuclear Research Centre, Mol, Belgium; 2 Royal Meteorological Institute of Belgium, Brussels, Belgium; 3 Department of Physics and Astronomy, Ghent University, Ghent, Belgium

## Abstract

On 6 January 2016, the Democratic People’s Republic of Korea announced to have conducted its fourth nuclear test. Analysis of the corresponding seismic waves from the Punggye-ri nuclear test site showed indeed that an underground man-made explosion took place, although the nuclear origin of the explosion needs confirmation. Seven weeks after the announced nuclear test, radioactive xenon was observed in Japan by a noble gas measurement station of the International Monitoring System. In this paper, atmospheric transport modelling is used to show that the measured radioactive xenon is compatible with a delayed release from the Punggye-ri nuclear test site. An uncertainty quantification on the modelling results is given by using the ensemble method. The latter is important for policy makers and helps advance data fusion, where different nuclear Test-Ban-Treaty monitoring techniques are combined.

## Introduction

The Comprehensive nuclear Test-Ban-Treaty (CTBT) has been open for signature since 1996 by the United Nations and aims to ban atmospheric, underwater and underground nuclear explosions. Although the Treaty has not yet entered into force, preparations are being made on the scientific, technical and political level. Compliance with the Treaty will be observed by the International Monitoring System (IMS). The IMS will measure infrasound, hydroacoustic and seismic waves globally to detect atmospheric, underwater and underground explosions. To discriminate nuclear explosions from conventional explosions, 80 stations will measure airborne radionuclides. To date, 84% of the network has been installed. Furthermore, the Treaty foresees the possibility to conduct on-site inspections once it has entered into force.

At least half of the 80 stations that will measure airborne radionuclide particles, will also be able to detect certain radioactive noble gases (^131m^Xe, ^133m^Xe, ^133^Xe and ^135^Xe, further on called radioxenon) that are created during a nuclear explosion. These noble gases are of interest since they are more likely to be released to the atmosphere after an underground or underwater nuclear explosion compared to particulates and reactive gasses. Additionally, they are not subject to deposition or chemical reactions in the atmosphere, which is beneficial from both an observational and modelling point of view. Finally, the half-lives of these isotopes are long enough to be captured by the IMS radionuclide stations, but short enough to avoid accumulation in the atmosphere over time (^131m^Xe: 11.84 d, ^133m^Xe: 2.20 d, ^133^Xe: 5.25 d and ^135^Xe: 9.14 h)^1^.

**Figure 1:**
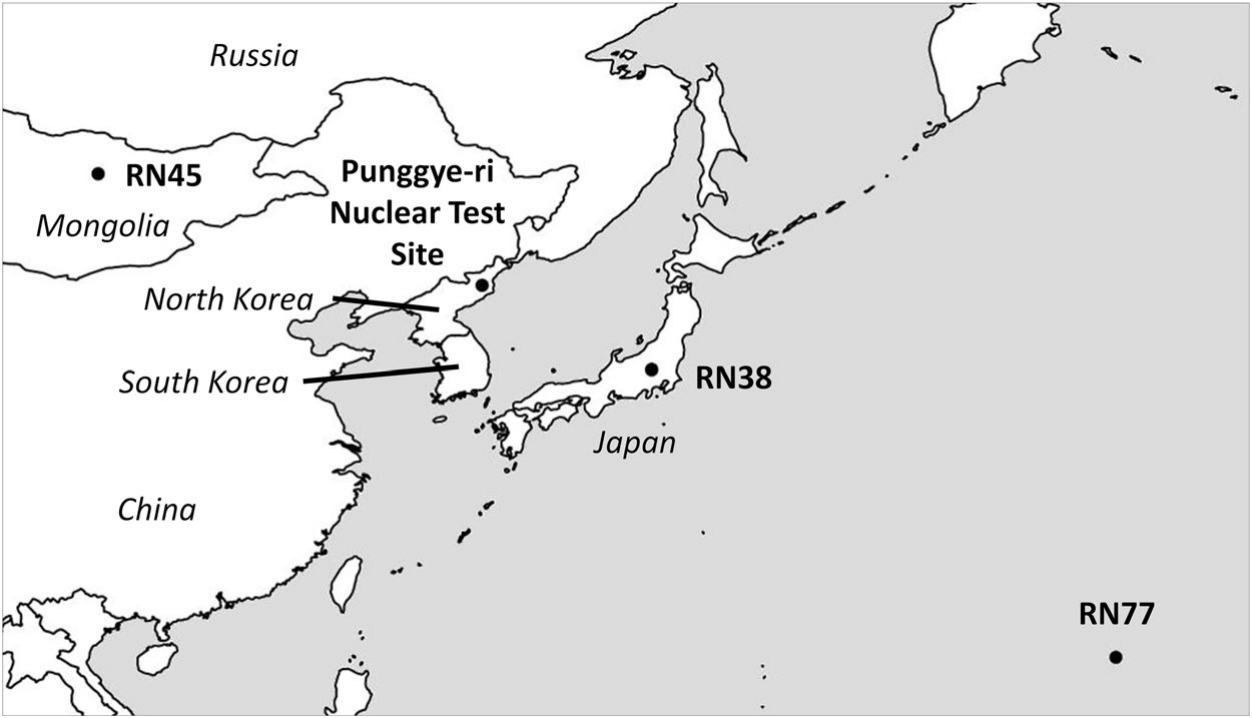
Location of the IMS stations and the Punggye-ri nuclear test site (bullets; text in bold), and the names of the nearby countries (in italic). The map has been generated using ref. 48.

If radioxenon is observed by the noble gas component of the IMS stations, atmospheric transport models can help with the determination of the location, release period and release amount of the source. In the past, radioxenon signatures have been linked to previous nuclear tests conducted by the DPRK^2–4^. However, radioxenon is not only produced during nuclear explosions. Civil sources such as medical isotope production facilities^5, 6^ (MIPFs) and nuclear power plants^7, 8^ (NPPs) also release radioxenon. These civil sources create a background of radioxenon that can be measured by the network^9, 10^ and could mask the signatures of a nuclear explosion^11^. Elevated ^133^Xe concentrations were measured at RN38 (Takasaki, Japan; see Fig. 1) in the past, when no nuclear tests were known to have been conducted by the DPRK. In 2015, six events occurred where ^133^Xe concentrations exceeded 1 mBq/m³ (for comparison: in 2015, the median activity concentration at RN38 was 0.151 mBq/m³ and the 95% quantile was 0.547 mBq/m³), but none of these could be linked to the DPRK.

On 6 January 2016, the DPRK announced to have conducted its fourth nuclear test. Seismic signals picked up by the IMS have shown that a man-made explosion occurred around 1:30~UTC at the Punggye-ri nuclear test site in the DPRK^12^. Since the explosion took place underground, it is not sure if and what amount of radioactivity has been released to the atmosphere - assuming the explosion was nuclear. Six weeks later, elevated ^133^Xe concentrations were measured at the IMS noble gas station RN38 (Supplementary Table S1). Besides RN38, two other nearby certified IMS stations (RN45, Ulaanbaatar, Mongolia and RN77, Wake Island, United States of America) were operational during that period. The locations of these IMS stations and the Punggye-ri nuclear test site in the DPRK are shown in Fig. 1.

## Inverse modelling

Inverse atmospheric transport modelling^13, 14^ plays an important role in determining source locations and emissions. Recently, inverse modelling has been used to estimate the release of airborne radionuclides after the Fukushima Dai-ichi nuclear power plant accident^15–19^. One of the challenges of inverse modelling is how to quantify uncertainty. Such an uncertainty quantification on the assessment of the signals of a possible nuclear explosion is highly desired, not only as a “good science” practice, but also for policy makers.

The inverse modelling technique involves finding a source term *Q(x, y, z, t)* that can best explain the observed concentrations *conc(x*_*0*_ *, y*_*0*_ *, z*_*0*_ *,t*_*start*_ *, t*_*stop*_ *)* measured at (x_0_, y_0_, z_0_) between times t_start_ and t_stop_ (Methods). Both the release and detections are assumed to take place in the lowest model layer of Flexpart, extending from the surface to 100 m above. Furthermore, we assume that a single point source with releases bounded between Q_min_ and Q_max_ is responsible for the observed concentrations (Methods). Once such an optimal source has been found, the corresponding concentrations can be calculated and compared with the observed concentrations. To quantify the deviation between the measured concentrations and the calculated concentrations that come from the optimal source term *Q(x, y, z, t)*, a quadratic cost function is used:

1 $$\,cost\,function=\sum _{samples}{(obs-calc)}^{2}$$

We select a grid box and assume it is the source location. We then apply inverse modelling to find a source term and quantify how well that grid box source can explain the observations by using Eq. 1. We repeat this procedure for all grid boxes in the lowest model layer of Flexpart. The method has been validated for three fictitious sources (Supplementary Figs S1, S2 and S3). The validation suggests that a short release leads to more network ambiguity, since fewer signals can be captured by the IMS stations (Methods).

## Possible source regions

We have applied the inverse modelling technique to the elevated ^133^Xe concentrations measured at RN38. The inverse modelling should make use of as much as possible observations to decrease network ambiguity^13^. However, several civil sources all over the world leave their mark on the network^9, 10^, so that the inclusion of observations outside the region of interest or outside the time period considered could lead to worse results. With these considerations in mind, we have selected 50 observations from three certified IMS noble gas stations (RN38, RN45 and RN77; Fig. 1). The ^133^Xe detections by the IMS stations used in the inverse modelling are listed in Supplementary Table S1. At station RN45, radioxenon activity concentration is measured during a 24-h period using the Spalax system^20, 21^. Stations RN38 and RN77 are equipped with the Sauna system^22^ and measure radioxenon activity concentration twice a day during a 12~h period. Non-detections were set to 0 mBq/m³ although the true value lies between 0 mBq/m³ and the minimum detectable concentration which is of the order 0.1 mBq/m³. The results are insensitive to this treatment of the non-detections; furthermore, the results are robust for a different selection of observations, except for the five samples taken at RN38 that contained most ^133^Xe.

The atmospheric transport and dispersion simulations were realized with the Lagrangian particle model Flexpart^23–25^ in backward mode^26^. As input, we have used 51 members of the ensemble variational data assimilation system^27, 28^ (EDA) of the European Centre for Medium-Range Weather Forecasts (Methods) with horizontal grid spacings of 0.5°. For every EDA member, a new Flexpart run was started. We have obtained 51 sources and corresponding cost function values for each grid point.

**Figure 2:**
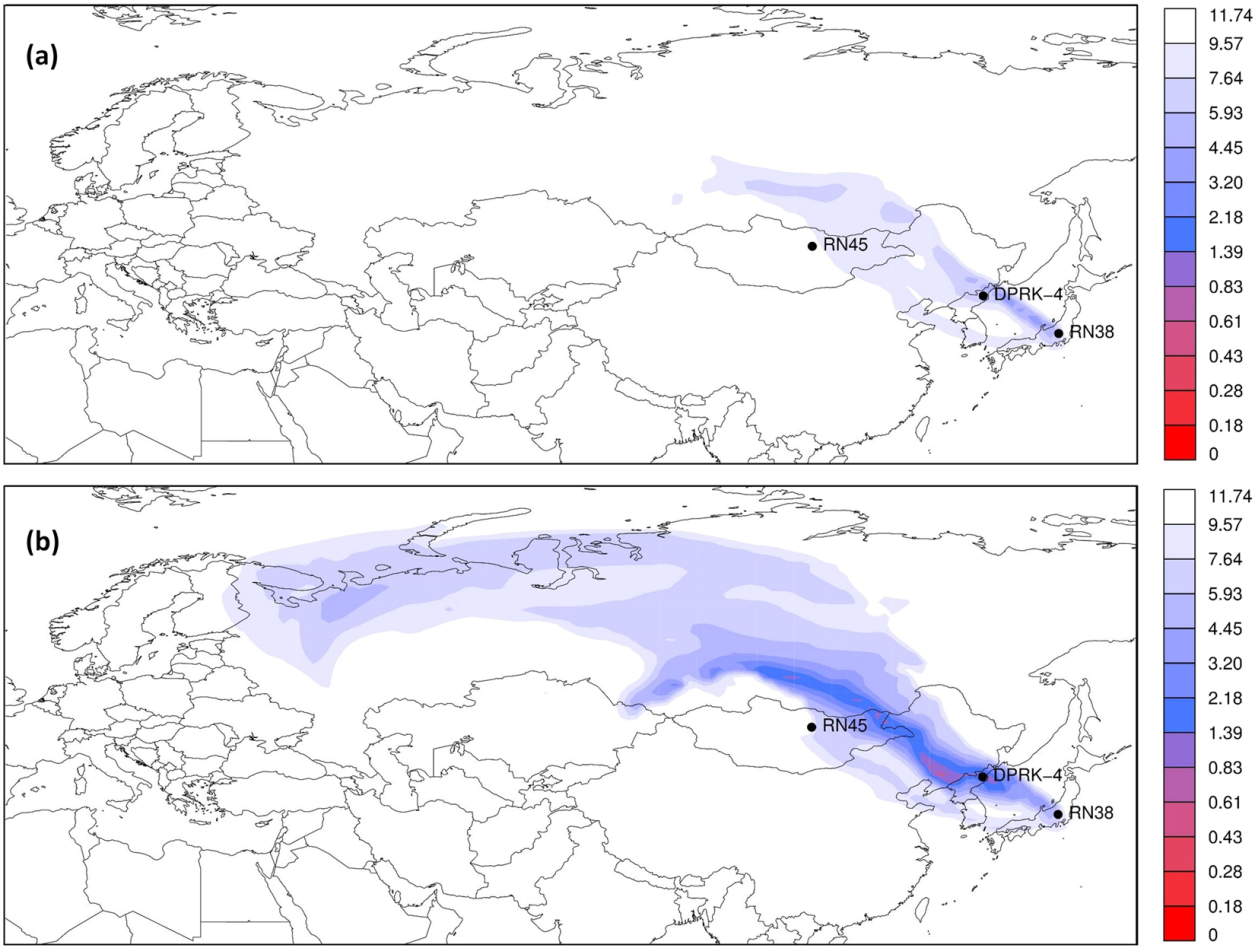
Map showing how well a grid box source can explain the observed ^133^Xe concentrations considered in the simulations (lower values denote a better match). Results are for (**a**) Q_max_ = 10^12^ Bq/day and (**b**) Q_max_ = 10^13^ Bq/day. The numbers in the legend are the square root of the cost function (Eq. 1) and can thus be seen as a RMSE (mBq/m³). Maps have been generated using ref. 48.

For the inverse modelling, two hypotheses are considered: the xenon originated from (i) a single civil source and (ii) the Punggye-ri test site where an underground explosion took place six weeks earlier. For the first hypothesis, we take Q_max_ = 10^12^ Bq/day, which is a reasonable assumption for the maximum release of civil sources in Eurasia^5^. For the second hypothesis, we increase Q_max_ to include possibly high radioxenon releases from an underground nuclear explosion. The ^133^Xe inventory associated with a 10 kton nuclear explosion is about 5 * 10^14^ Bq after 41 days of decay^29^. For the simulations, we take Q_max_ = 10^13^ Bq/day since it is unlikely that all available ^133^Xe would be released at once after an underground nuclear explosion. Maps of the cost function values for both hypotheses are shown in Fig. 2 (the median of the 51 ensemble members is shown).

For the civil source assumption, the lowest values of the cost function can be found in a narrow area northwest of RN38 up to the northeast of the People’s Republic of China (Fig. 2a). However, the cost function values in these grid points are rather high, which indicates that the current assumptions do not allow a solution that is compatible with the observations. Therefore, we exclude the possibility that a single civil source with emissions not higher than 10^12^ Bq/day was responsible for the observed ^133^Xe concentration.

For the nuclear explosion assumption (Fig. 2b), a much larger area with lower cost function values appears. A narrow band with low cost function values starting in the DPRK goes northwest into China, along the border with Mongolia, and finally into Russia. However, no civil sources in that area are known to the authors that could emit such an amount of xenon (also, see for instance Fig. 1 of ref. 10). The Punggye-ri nuclear test site is a possible source since the cost function is low in that area.

## Pointwise probability maps

The inverse modelling (Fig. 2) discriminates between regions that are compatible with the observations (of which the area depends on the network ambiguity, the source term and the meteorological conditions), and regions that are not compatible with the observations. An uncertainty estimate is desired for such an analysis, for which we have used an ensemble. To allow uncertainty visualization, a threshold to the cost function has been defined (Methods). We now define (im)possible source regions as grid boxes where more than 95% (less than 5%) of the members have cost functions below the threshold (see subsection Uncertainty visualisation in Methods). No confident statement can be made about grid boxes where more than 5% but less than 95% of the members have cost functions below the threshold: it is less likely that the true source lies in that region, but it cannot be excluded.

**Figure 3:**
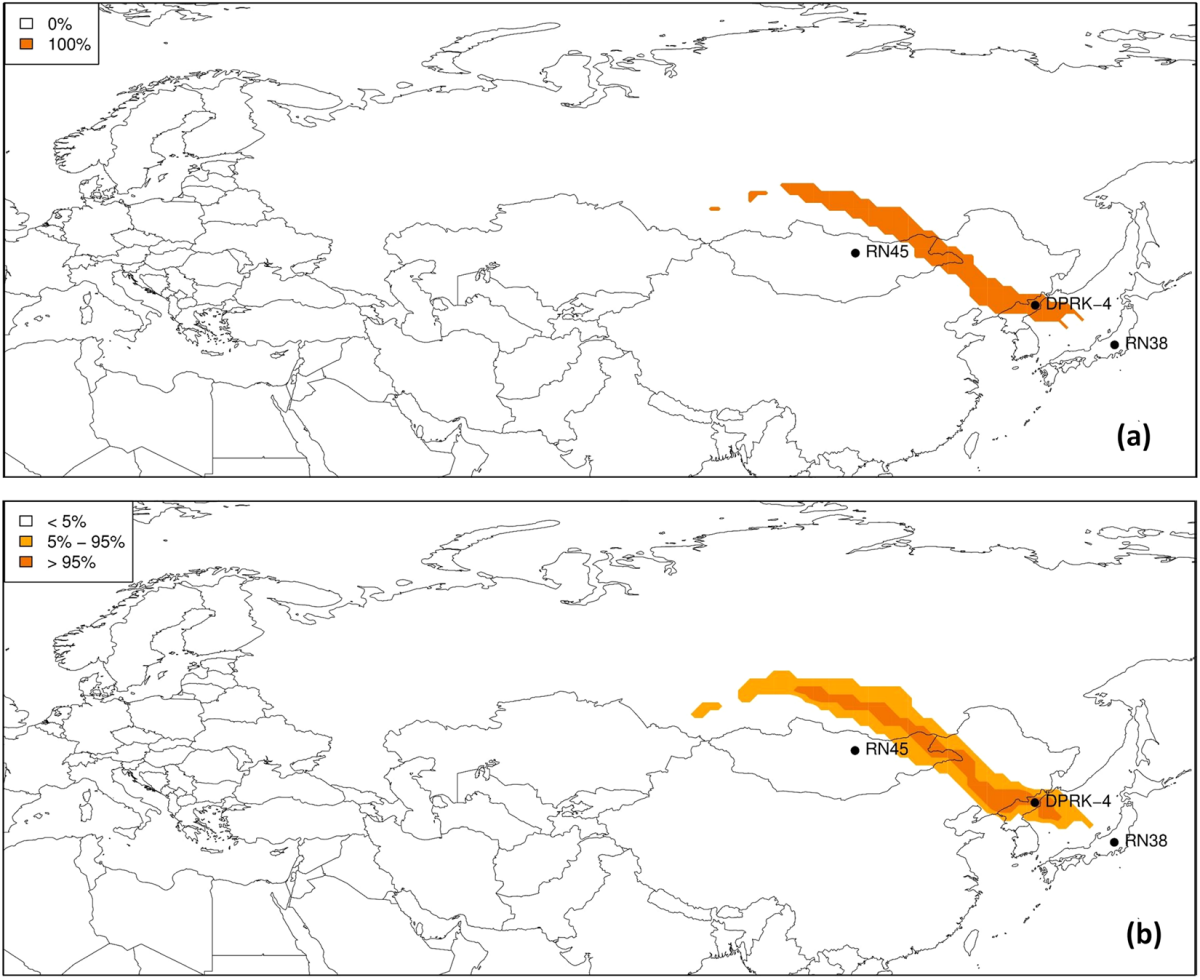
Pointwise probability that a certain grid point is a possible source for (**a**) the unperturbed simulation (thus fully deterministic) and (**b**) the full 51 member ensemble. Maps have been generated using ref. 48.

The results are shown in Fig. 3 for the deterministic, unperturbed simulation only and the full ensemble, both for Q_max_ = 10^13^ Bq/day. Note that for the deterministic simulation (Fig. 3a), the grid box cost function is below the threshold with either 0% or 100% probability. Compared with the deterministic simulation, the ensemble (Fig. 3b) narrows the area of possible sources. On the other hand, a region appears where the percentage of members below the threshold ranges between 5% and 95%. This results from the perturbations in the meteorological fields, so that for different members, different grid boxes become possible sources. These maps allow to quickly identify which sources are compatible with the observations used in the inverse modelling. The Punggye-ri nuclear test site falls within the region of possible sources (Fig. 3).

## Possible release at the Punggye-ri site

**Figure 4:**
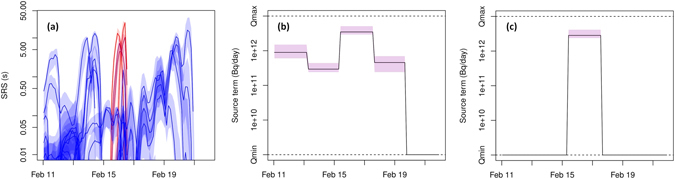
(**a**) SRS values at the Punggye-ri nuclear test site for each sample considered in the inverse modelling as a function of time; colours are semi-transparent and proportional to the sample’s observed ^133^Xe concentration (blue: lowest concentration; red: highest concentration). (**b**) Possible ^133^Xe release at the Punggye-ri site as a function of time. (**c**) Possible ^133^Xe release at the Punggye-ri site as a function of time but with all samples equal to or below 0.3 mBq reset to zero. The shadings represent values within the 2.5% and 97.5% quantile of the ensemble.

From the analysis above, we assume that the ^133^Xe observed at RN38 originated from the Punggye-ri nuclear test site. Here, we assess such a possible release. Figure 4(a) shows the source-receptor-sensitivity^26, 30^ (SRS) values at the Punggye-ri nuclear test site for all 50 samples with the corresponding uncertainty obtained from the ensemble. The colours in Fig. 4a denote the amount of ^133^Xe in the sample (blue: no ^133^Xe, red: high level of ^133^Xe). The SRS values corresponding with the elevated radioxenon samples taken at RN38 (shown in red in Fig. 4a) form a narrow and coherent period, so that a single release can explain the observations. There are other samples with non-zero SRS during that period containing few or no radioxenon, but this does not pose an inconsistency since the SRS values are two orders of magnitude lower, resulting in ^133^Xe concentrations too low to be detected. Therefore, a release at the Punggye-ri nuclear test site is not contradicted by the absence of high radioxenon concentrations at other times or locations than what has been observed.

Figure 4(b) shows the optimal source term and its uncertainty obtained from the inverse modelling at the Punggye-ri nuclear test site. There is a peak in the ^133^Xe release of 3.8 * 10^12^ Bq/day that coincides with the non-zero SRS values of the elevated radioxenon samples at RN38. After that peak release, the ensemble shows that there could have been a small release of up to 5 * 10^11^ Bq/day or even no (significant) release at all. The days before the peak, the inverse modelling suggests that there could have been a ^133^Xe release of about 10^12^ Bq/day.

As already stated, civil sources also emit radioxenon that can be detected by the IMS noble gas stations network^9, 10^. Since it is very challenging to accurately simulate the radioxenon background^31–33^, we simply assume that all observed ^133^Xe concentrations equal to or below 0.3 mBq/m^3^ are coming from one or multiple civil sources. These concentrations were reset to zero, but were still used in the inverse modelling; the other ^133^Xe concentrations are decreased with 0.3 mBq/m^3^. A value of 0.3 mBq/m³ was chosen, since this corresponds roughly with the average activity concentration measured at IMS stations (see for instance Fig. 4 of ref. 10). Although the radioxenon background has a strong spatial and temporal variability, we have chosen to use one number for practical reasons. By reapplying the inverse modelling, the same though slightly weaker ^133^Xe peak release is found, without any significant releases before or after the peak (Fig. 4c). We have recalculated the cost function and find a similar pattern as in Fig. 2 (not shown). The accumulated ^133^Xe release in Fig. 4c ranges between 5.4 * 10^12^ Bq and 9.3 * 10^12^ Bq (obtained from the 2.5% and 97.5% quantile of the ensemble), with a median of 6.78 * 10^12^ Bq. Without the background correction, the 97.5% quantile is 1.1 * 10^13^ Bq. Taking into account 41 days of decay, a crude estimate of the minimal initial ^133^Xe inventory is 1.5 * 10^15^ Bq, which is about 1.3% of the full ^133^Xe inventory for a 10 kton nuclear explosion (the given value is based on the ensemble median).

## Absence of ^131m^Xe detections

The presence of other radioxenon isotopes such as ^131m^Xe allow to study radioxenon isotopic ratios with the aim to discriminate between a nuclear explosion and civil sources^34^. However, no other radioxenon isotopes have been observed in the samples containing high amounts of ^133^Xe. Given the shorter half-lives of ^133m^Xe and ^135^Xe, only ^131m^Xe is eligible for detection, besides ^133^Xe. We now assess whether the non-detection of ^131m^Xe is compatible with a possible release of ^131m^Xe at the Punggye-ri site.

From the calculated minimum ^133^Xe inventory (based on the ensemble median) at the Punggye-ri site, we can estimate the minimum ^131m^Xe inventory. We take a cumulative fission yield of 0.06%^29^. We assume an identical release fraction for ^131m^Xe as for ^133^Xe. The estimated ^131m^Xe release is 7.0 * 10^11^ Bq. The calculated ^131m^Xe activity concentrations at RN38 are listed in Table 1. Results show that non-detections are not unlikely since the calculated ^131m^Xe fall below the minimum detectable concentration.

**Table 1:** Calculated ^131m^Xe activity concentration values for the IMS noble gas station RN38 and the station’s Minimum Detectable Concentration (MDC) from IMS observations.

Collection start (UTC)		Collection stop (UTC)		Activity (mBq/m³)	MDC (mBq/m³)
20160216	20:35:00	20160217	08:35:00	0.20	0.28
20160217	08:35:00	20160217	20:35:00	0.24	0.29
20160217	20:35:00	20160218	08:35:00	0.16	0.27
20160218	08:35:00	20160218	20:35:00	0.12	0.26
20160218	20:35:00	20160219	08:35:00	0.02	0.22

## Discussion

We have assessed whether the Punggye-ri site is a plausible source of the elevated ^133^Xe concentrations observed by the IMS noble gas station RN38 six weeks after the announced nuclear test by the DPRK. Results show that no single civil source, operating under normal circumstances, can explain all radioxenon detections and non-detections. Although we cannot exclude the possibility that a local civil source^35^ is responsible for the observed xenon at RN38 (since our analysis dealt with long-range modelling), it is unlikely since high xenon concentrations were detected in five consecutive samples rather than in a single sample. On the other hand, results are consistent with a release from the Punggye-ri nuclear test site in the DPRK (Fig. 2). An ensemble is used to construct pointwise probability maps such as Fig. 3. By assuming that a ^133^Xe release took place at the Punggye-ri site, we have calculated the corresponding source term (Fig. 4). Taking into account radioactive decay, we have estimated the minimal initial ^133^Xe inventory to range between 1.2 * 10^15^ Bq and 2.1 * 10^15^ Bq (2.5% and 97.5% quantile), which corresponds to about 1.3% of the full inventory of a 10 kton nuclear explosion.

**Figure 5:**
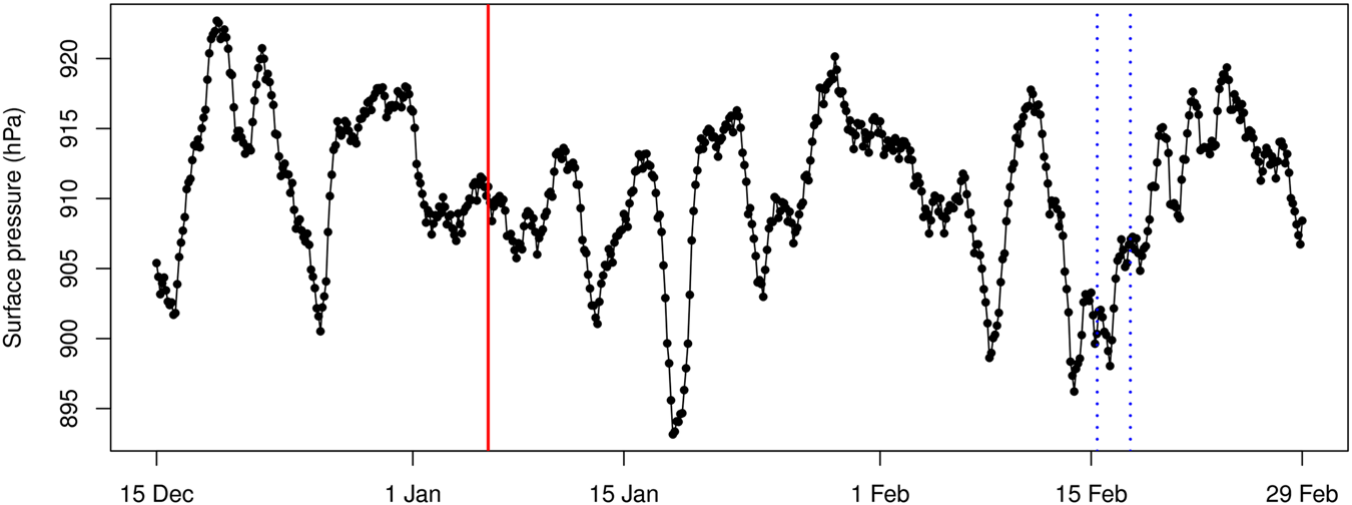
Surface pressure at the Punggye-ri nuclear test site obtained from ECMWF analyses. The full red vertical line shows the timing of the DPRK underground explosion, the dotted blue vertical lines show the possible release period for the Punggye-ri nuclear test site.

Two scenarios could have caused the delayed release from the underground cavity. The first scenario is human intervention such as opening of tunnels, drilling of holes. The second scenario involves gas transport mechanisms that can cause radioxenon to move towards the surface of the soil. These processes are pressure driven from the cavity, multiphase convection and barometric pumping^36^. In the assessment of the third announced nuclear test by the DPRK, it was shown that the likely release at the Punggye-ri nuclear test site coincided with low atmospheric pressure^4^. It is not clear if and to what extent the low air pressure influenced the release of radioxenon, although the principle of atmospheric pumping is well known^36–39^. Although an assessment on the cause of the delayed xenon release is outside the scope of this paper, we plot the air pressure obtained from ECMWF analyses for the Punggye-ri site and see that the possible release period coincides with an episode of low air pressure (Fig. 5).

## Methods

### Data and models

The Lagrangian particle model Flexpart version 9.02 has been used for the atmospheric transport and dispersion simulations. Flexpart requires numerical weather prediction data as input, which have been extracted from the MARS server of ECMWF using the extraction software available at the Flexpart website^40^. Meteorological data are known to have the biggest impact on atmospheric transport and dispersion simulation uncertainty^41, 42^. We have used the analyses, i.e. the initial model states for the 51 members of the EPS from the ensemble variational data assimilation system (EDA) of ECMWF to quantify this uncertainty. EDA makes use of 4D variational assimilation (known as “4D-Var”) to combine observations and a short-range forecast in an optimal way and is run twice every day. The ensemble consists of 50 perturbed realisations or “members” and a control member, which is not perturbed. Perturbations are generated by perturbing the assimilated observations^27, 28^, the sea-surface temperature field^43^ and the model physics tendencies^44^. By construction, every member is equally likely to occur and the spread between the members contains information about the uncertainty of the simulation. The model extends to a depth of 0.01 hPa and has 137 vertical hybrid σ-levels, which follow the earth’s surface in the lower-most troposphere, but gradually follow surfaces of constant pressure aloft. Three-hourly global model data with 0.5° horizontal grid spacing have been used for the Flexpart simulations. The boundary layer height is calculated in Flexpart using the critical Richardson number^25, 45^.

### Inverse modelling method

The relevant radioxenon sources (nuclear power plants, medical isotope production facilities and, potentially for this case, releases from a nuclear test site) can be considered as point sources for long-range atmospheric transport problems. It is well known that the concentrations in any geotemporal point are, most of the time, dominated by a single source lying upstream on the trajectory of the atmospheric transport. Furthermore, the Punggye-ri test site is remote from known civil sources, so that its plume can remain relatively distinct^46^. As a consequence, we assume that the elevated radioxenon observations are originating from a single grid box source. For every grid box (x_0_, y_0_, z_0_) in the lowest model layer, the cost function in Eq. (1) is minimized as a function of the source term Q(x, y, z, t) = Q(t) * δ(x − x_0_, y − y_0_, z − z_0_). This is done with a quasi-Newton method (we have used the R Statistical Software routine *nlminb* ^47^). In Flexpart, the concentrations scale linearly with the source, so there is no need to call Flexpart for every iteration of the minimization.

The source term is bounded between a minimum and maximum value Q_min_ and Q_max_, the latter to prevent unrealistically high source terms. The minimum release Q_min_ is equivalent to Q_min_ = 0 Bq/h for long-range atmospheric transport due to the large dilution factors in the atmosphere and the noble gas station minimum detectable concentration.

Mind that the transport between the Punggye-ri test site and the IMS stations (Fig. 1) takes place at the synoptic scale. The time resolution of Q(t) has been limited by averaging the SRS fields to a period of roughly 2 days. This is sufficient to resolve the synoptic-scale signal, and ensures that we have an overdetermined problem.

### Validation with fictitious sources

The inverse modelling method using observations from certified IMS noble gas stations has been validated by reconstructing three fictitious sources at the Punggye-ri nuclear test site in mid-February 2016, using the deterministic model. Perfect pseudo-observations were constructed with Flexpart in forward mode. Since Flexpart is self-adjoint^26^, the SRS fields obtained by running Flexpart in backward mode used for the inverse modelling can also be considered as perfect. The concentrations at receptor stations were averaged over 12 h and 24 h time periods corresponding to the collection period of the IMS stations^20–22^, and rounded to 0.1 mBq/m³ (roughly the absolute uncertainty on the measured ^133^Xe concentrations). This implies a loss of information, and therefore, despite the use of perfect SRS fields and quasi-perfect observations, no exact match can be expected between the calculated source and the true source. In total, 50 pseudo-observations have been constructed for the stations RN38, RN45 and RN77.

For each fictitious source, we have assessed whether (i) the Punggye-ri nuclear test site is a possible source and (ii) given the correct source location, the true source term can be reconstructed. For the optimisation, we have used the bounds Q_min_ = 5 * 10^9^ Bq/h and Q_max_ = 5 * 10^12^ Bq/h. For each fictitious source, three different source terms have been calculated: (i) a time independent source term (1 unknown), (ii) a time dependent source term with intervals of roughly two days (5 unknowns) and (iii) a time dependent source term with intervals of roughly one day (10 unknowns). Results are shown in Supplementary Figs S1, S2 and S3. Note that the time resolution of the true source and the source in the inverse modelling differ, so that no exact match can be found for the second and third fictitious source.

The first fictitious source is a continuous ^133^Xe release of 10^11^ Bq/h. We have applied the inverse modelling method assuming a time-independent source term (Supplementary Fig. S1) and find a quasi-exact match between the modelled and prescribed source term. When the number of unknowns is increased by allowing the source term to vary with time, the source found by the inverse modelling deviates from the true source (Supplementary Fig. S1). Note, however, that the square root of the cost function values (which can be interpreted as a RMSE) decreases only slightly compared to the constant release assumption. The grid boxes with the lowest RMSE explain best the observations. We see a narrow area roughly northwest of the IMS station RN38 where the RMSE is low (Supplementary Fig. S1). Very low values are found in the region around the Punggye-ri nuclear test site, and it is clearly a possible source. When introducing more time dependency in the calculated source, other regions become possible sources as can be seen by the low RMSE (Supplementary Fig. S1). This ambiguity, that is, the fact that multiple grid boxes have low RMSE for the given measurements, can be attributed to the geotemporal resolution of the time sampling frequencies and the locations used in the IMS noble gas network.

The second fictitious ^133^Xe source varies with time and its release changes step-wise with a maximum halfway during the release period. This time-dependent source cannot be captured well by a continuous source assumption (Supplementary Fig. S2). As a consequence, the Punggye-ri nuclear test site does not show up as a possible source as can be seen from the large RMSE (Supplementary Fig. S2). However, it is worth noting that areas further upwind do show up as possible sources. When the source obtained from the inverse modelling method is allowed to vary with time, a quite good agreement is obtained (Supplementary Fig. S2). At the same time, the Punggye-ri nuclear test site is now a possible source (Supplementary Fig. S2). This case highlights the importance of time dependency when reconstructing a time varying source, both for the source term as for the source location determination.

The third fictitious source is a short ^133^Xe release of 10^11^ Bq/h. A constant release is not suited to match the true source term, and as a result the true source location has a large RMSE (Supplementary Fig. S3). Allowing the source to vary with time results in a good fit with the true source. The Punggye-ri nuclear test site is now shown as a possible source, although a large area northwest of the DPRK has also a very low RMSE. This ambiguity, resulting from limitations in the network’s geotemporal resolution, is most pronounced for this fictitious source. This is likely due to the shortness of the release period, so that a shorter signal could be captured by the IMS stations.

For the three cases considered, this method is able to identify a possible source region around the true source if the reconstructed source term is allowed to vary with time. For a known source location, this method allows one to successfully reproduce the source term, although with limited temporal resolution to avoid a noisy solution. A quantitative evaluation of the inverse modelling for the different fictitious sources is given in Table 2. It is concluded that five intervals of roughly two days in the reconstructed source give good results since it provides sufficient time resolution, without making the reconstructed releases noisy. The inverse problem is more complex with real data since (i) SRS fields contain errors and (ii) observations are contaminated by the radioxenon background from civil sources.

**Table 2:** Minimum cost function value of the full grid, cost function value at the Punggye-ri nuclear test site and distance between both. The last row shows the quantile of the Punggye-ri cost function compared to all grid boxes in the Eurasia domain (9211 grid points). Values are given for the three fictitious sources and different time resolutions of the source term Q(t). The best values for each test are shown in bold

	test 1			test 2			test 3		
# Q(t) intervals	1	5	10	1	5	10	1	5	10
Min cost function	0.212	0.067	0.029	19.49	3.70	0.69	0.131	0.011	0.001
Punggye-ri cost function	0.940	0.318	0.184	196.4	24.6	20.9	2.307	0.120	0.104
Distance (km)	309	**307**	**307**	1110	**299**	**299**	1735	**299**	1760
Punggye-ri quantile	**0.999**	**0.999**	**0.999**	0.909	**0.991**	0.990	0.982	**0.997**	0.994

.

### Uncertainty visualisation

To allow uncertainty visualisation of the cost function maps, as shown in Fig. 3, a cost function threshold has been defined using Eq. 2 with an absolute error β = 0.2 mBq/m³ (roughly corresponding to the minimum detectable concentration, important in case nothing is detected). The relative error α is chosen to be 20%, since this confines the area of possible sources without being too strict.

2 $$threshold=\sum _{samples}{(\alpha \ast obs+\beta )}^{2}$$

### Data Availability

The datasets generated during and/or analysed during the current study are available from the corresponding author on reasonable request.

## Electronic supplementary material

Supplementary information (Figures and Table)
