# Supplementary material for: Assessment of the announced North Korean nuclear test using long-range atmospheric transport and dispersion modelling
Source: Sci Rep. 2017 Aug 18;7:8762. doi: 10.1038/s41598-017-07113-y (PMC5562919; doi:10.1038/s41598-017-07113-y)
Supplement: Supplementary file 1 — Supplementary information (Figures and Table) [file 41598_2017_7113_MOESM1_ESM.pdf]

SUPPLEMENTARY INFORMATION to “Assessment of the announced North Korean nuclear test using long-range atmospheric transport and dispersion modelling”

Pieter De Meutter[1,2,3,*], Johan Camps[1], Andy Delcloo[2,3] and Piet Termonia[2,3]
[1]Belgian Nuclear Research Institute, Mol, Belgium
[2]Royal Meteorological Institute of Belgium, Brussels, Belgium
[3]Department of Physics and Astronomy, Ghent University, Ghent, Belgium

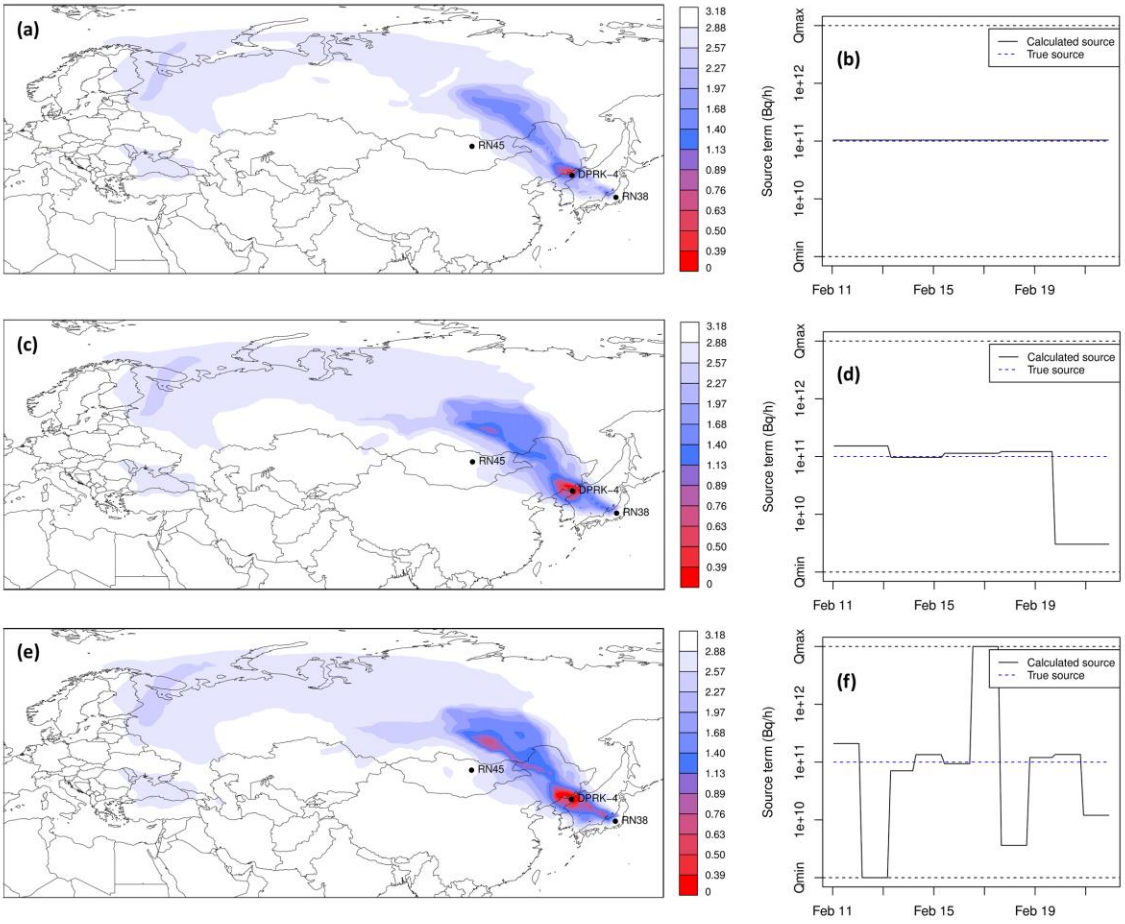

Supplementary Figure S1: (a, c, e) Map showing how well a grid box source can explain the observed ^{133}Xe concentrations considered in the simulations (lower values denote a better match). The numbers in the legend are the square root of the cost function (Eq. 1) and can thus be seen as a RMSE (mBq/m^3). (b, d, f) Modelled (solid black line) and true (dashed blue line) fictitious ^{133}Xe source term at the Punggye-ri nuclear test site. Results are shown for three different time resolutions of the source term (a-b: time-independent source term; c-d: source term with five time intervals of roughly 2 days; e-f: source term with ten time intervals of roughly 1 day). Maps have been generated using ref 48.

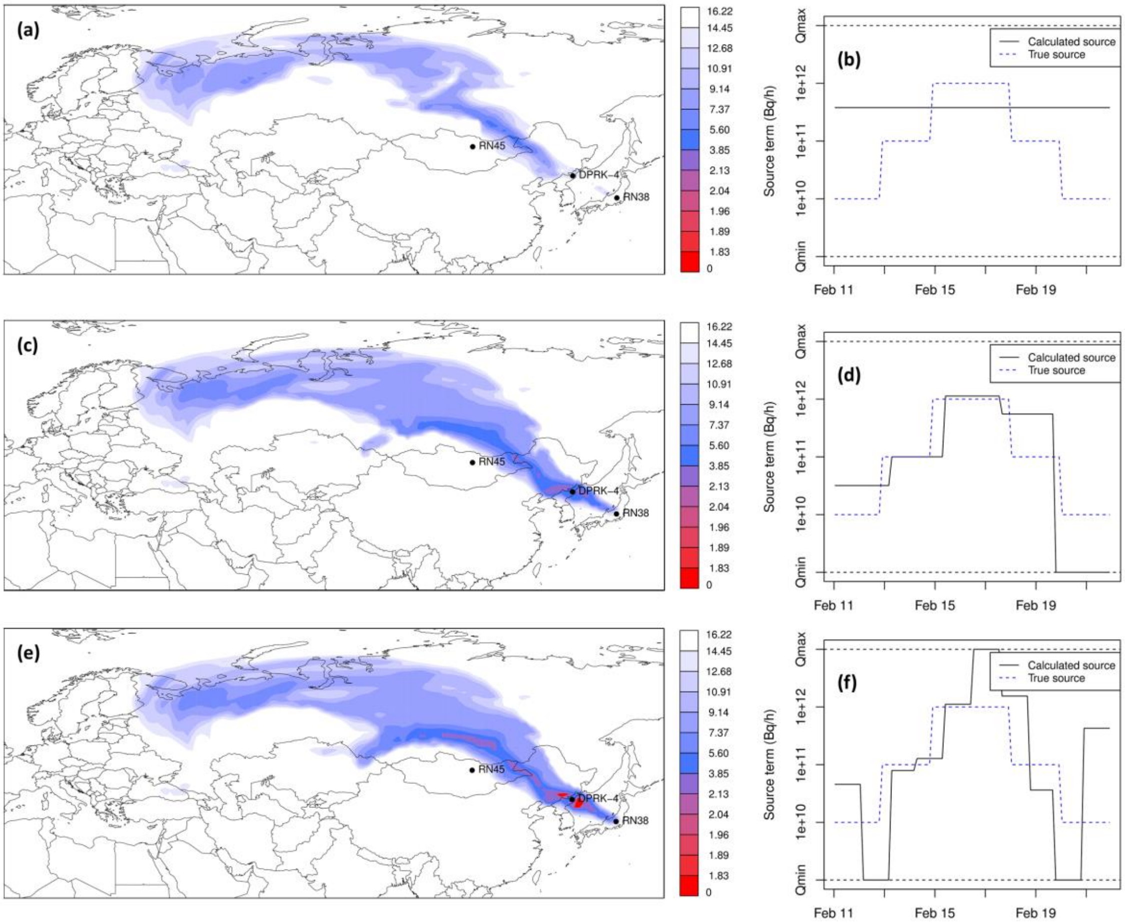

Supplementary Figure S2: As Supplementary Figure S1, but for a stepwise time-dependent fictitious source. Maps have been generated using ref 48.

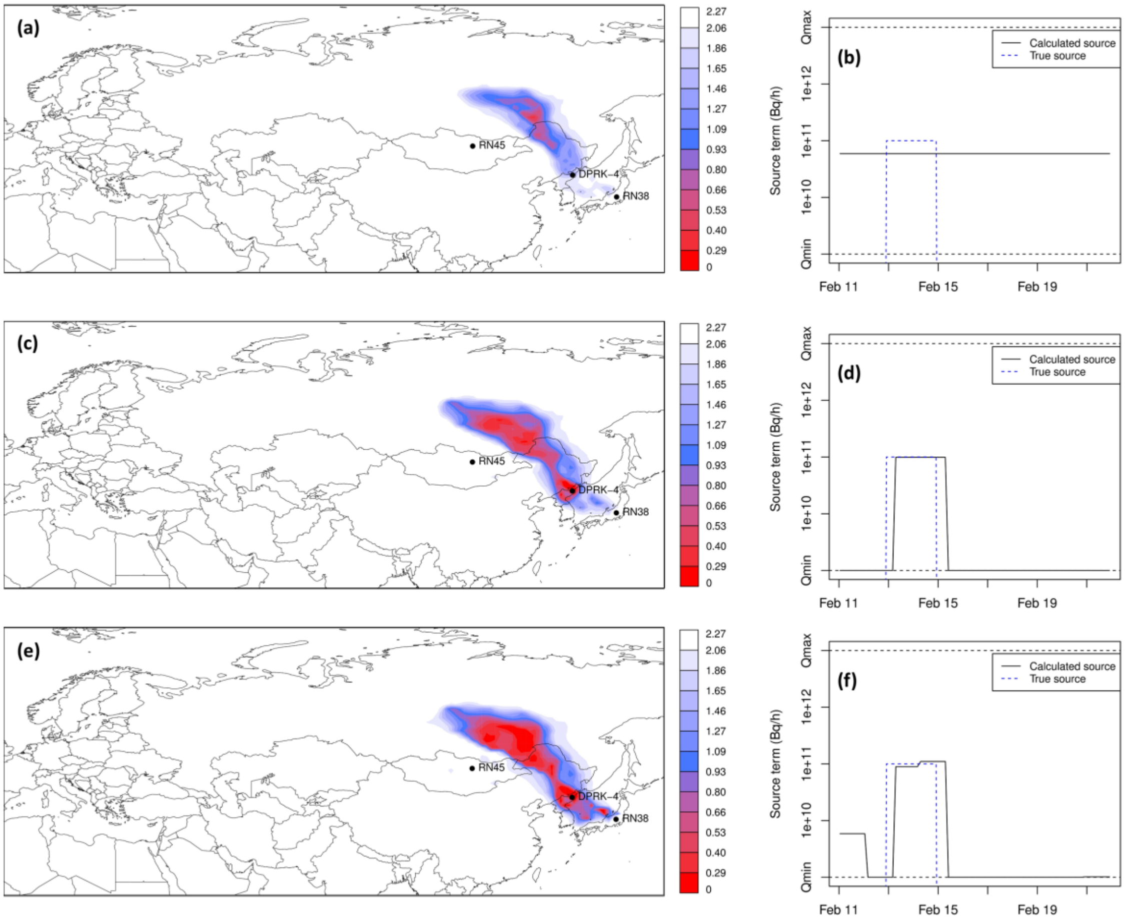

Supplementary Figure S3: As Supplementary Figure S1, but for a fictitious short release. Maps have been generated using ref 48.

Supplementary Table S1: ^{133}Xe activity concentrations observed by the IMS noble gas stations used for the inverse modelling.

Station	Collection start (UTC)		Collection stop (UTC)		Activity (*mBq/m³*)	Uncertainty (*mBq/m³*)
RN38	20160212	08:35:00	20160212	20:35:00	0.221	0.044
RN38	20160212	20:35:00	20160213	08:35:00	0.087	0.039
RN38	20160213	08:35:00	20160213	20:35:00	0.0	0.0
RN38	20160213	20:35:00	20160214	08:35:00	0.0	0.0
RN38	20160214	08:35:00	20160214	20:35:00	0.221	0.050
RN38	20160214	20:35:00	20160215	08:35:00	0.193	0.046
RN38	20160215	08:35:00	20160215	20:35:00	0.187	0.046
RN38	20160215	20:35:00	20160216	08:35:00	0.135	0.048
RN38	20160216	08:35:00	20160216	20:35:00	0.254	0.047
RN38	20160216	20:35:00	20160217	08:35:00	1.758	0.091
RN38	20160217	08:35:00	20160217	20:35:00	1.790	0.093
RN38	20160217	20:35:00	20160218	08:35:00	1.440	0.087
RN38	20160218	08:35:00	20160218	20:35:00	1.300	0.081
RN38	20160218	20:35:00	20160219	08:35:00	0.670	0.069
RN38	20160219	08:35:00	20160219	20:35:00	0.0	0.0
RN38	20160219	20:35:00	20160220	08:35:00	0.170	0.051
RN38	20160220	08:35:00	20160220	20:35:00	0.286	0.049
RN38	20160220	20:35:00	20160221	08:35:00	0.104	0.047
RN38	20160221	08:35:00	20160221	20:35:00	0.216	0.045
RN38	20160221	20:35:00	20160222	08:35:00	0.0	0.0
RN38	20160222	08:35:00	20160222	20:35:00	0.100	0.049
RN45	20160211	22:00:00	20160212	22:00:00	0.0	0.0
RN45	20160212	22:00:00	20160213	22:00:00	0.295	0.079
RN45	20160213	22:00:00	20160214	22:00:00	0.0	0.0
RN45	20160214	22:00:00	20160215	22:00:00	0.0	0.0
RN45	20160215	22:00:00	20160216	22:00:00	0.0	0.0
RN45	20160216	22:00:00	20160217	22:00:00	0.0	0.0
RN45	20160217	22:00:00	20160218	22:00:00	0.0	0.0
RN45	20160218	22:00:00	20160219	22:00:00	0.260	0.080
RN45	20160219	22:00:00	20160220	22:00:00	0.328	0.083
RN45	20160220	22:00:00	20160221	22:00:00	0.0	0.0
RN45	20160221	22:00:00	20160222	22:00:00	0.0	0.0
RN45	20160222	22:00:00	20160223	22:00:00	0.0	0.0
RN77	20160215	03:15:00	20160215	15:15:00	0.0	0.0
RN77	20160215	15:15:00	20160216	03:15:00	0.0	0.0
RN77	20160216	03:15:00	20160216	15:15:00	0.064	0.042

RN77	20160216	15:15:00	20160217	03:15:00	0.0	0.0
RN77	20160217	03:15:00	20160217	15:15:00	0.0	0.0
RN77	20160217	15:15:00	20160218	03:15:00	0.082	0.042
RN77	20160218	03:15:00	20160218	15:15:00	0.087	0.039
RN77	20160218	15:15:00	20160219	03:15:00	0.0	0.0
RN77	20160219	03:15:00	20160219	15:15:00	0.117	0.041
RN77	20160219	15:15:00	20160220	03:15:00	0.0	0.0
RN77	20160220	03:15:00	20160220	15:15:00	0.0	0.0
RN77	20160220	15:15:00	20160221	03:15:00	0.0	0.0
RN77	20160221	03:15:00	20160221	15:15:00	0.0	0.0
RN77	20160221	15:15:00	20160222	03:15:00	0.0	0.0
RN77	20160222	03:15:00	20160222	15:15:00	0.209	0.048
RN77	20160222	15:15:00	20160223	03:15:00	0.074	0.043
RN77	20160223	03:15:00	20160223	15:15:00	0.108	0.045
